# Changes in bacterial number at different sites of oral cavity during perioperative oral care management in gastrointestinal cancer patients: preliminary study

**DOI:** 10.1590/1678-7757-2017-0516

**Published:** 2018-05-29

**Authors:** Tomoko KAWANO, Hideo SHIGEISHI, Eri FUKADA, Takamichi YANAGISAWA, Nobukazu KURODA, Toshinobu TAKEMOTO, Masaru SUGIYAMA

**Affiliations:** 1 Hiroshima University, Graduate School of Biomedical & Health Sciences, Program of Oral Health Sciences, Department of Public Oral Health, Hiroshima, Japan.; 2 Takarazuka Municipal Hospital, Department of Dentistry & Oral Surgery, Takarazuka, Japan.; 3 Takarazuka Municipal Hospital, Department of Surgery, Takarazuka, Japan.; 4 Hiroshima University, Graduate School of Biomedical & Health Sciences, Program of Oral Health Sciences, Department of Oral Health Management, Hiroshima, Japan.

**Keywords:** Oral health, Perioperative care, Bacterial count, Gastrointestinal cancer

## Abstract

**Objective:**

The objective of this study was to clarify differences in bacterial accumulation between gastrointestinal cancer patients who underwent severely invasive surgery and those who underwent minimally invasive surgery.

**Material and Methods:**

We performed a preliminary investigation of gastrointestinal cancer patients who were treated at the Department of Surgery, Takarazuka Municipal Hospital, from 2015 to 2017 (n=71; 42 laparoscopic surgery, 29 open surgery) to determine changes in bacterial numbers at different sites of the oral cavity (tongue dorsum, gingiva of upper anterior teeth, palatoglossal arch), as well as mouth dryness and tongue coating indices. Specifically, patients received professional tooth cleaning (PTC), scaling, tongue cleaning, and self-care instruction regarding tooth brushing from a dental hygienist a day before the operation. Professional oral health care was also performed by a dental hygienist two and seven days after surgery. Oral bacteria numbers were determined using a bacterial counter with a dielectrophoretic impedance measurement method.

**Results:**

The number of bacteria at all three examined sites were significantly higher in the open surgery group when compared to the laparoscopic surgery group on the second postoperative day. Relevantly, bacterial count in samples from the gingiva of the upper anterior teeth remained greater seven days after the operation in patients who underwent open surgery. Furthermore, the dry mouth index level was higher in the open surgery group when compared to the laparoscopic surgery group on postoperative days 2 and 7.

**Conclusions:**

Even with regular oral health care, bacterial numbers remained high in the upper incisor tooth gingiva in gastrointestinal cancer patients who received open surgery. Additional procedures are likely needed to effectively reduce the number of bacteria in the gingival area associated with the upper anterior teeth.

## Introduction

Performing dental treatments, such as periodontal and dental caries treatments, extraction of non-preservable teeth and replacement of missing teeth, are required before starting cancer treatment to reduce postoperative complications. In addition, perioperative professional oral health care is recognized to be important for cancer patients^18^ ^,^ ^26^.

Colorectal cancer is a predominant type of cancer and the third leading cause of cancer-associated death worldwide^1^, while gastric cancer is also one of the most common types of malignancies throughout the world^2^. Surgical resection is the primary procedure to treat patients with gastrointestinal cancer, with laparoscopic surgery being widely adopted for gastrointestinal cancers, such as gastric and colorectal cancer^10^ ^,^ ^20^. There are reports that perioperative oral care may improve respiratory function in patients who undergo gastrointestinal surgery by reducing the number of bacteria species^25^, indicating that perioperative oral health care may play a significant role in reducing post-operative complications in those patients.

We consider that gastrointestinal cancer patients undergoing severely invasive surgery (i.e., open surgery) may have worse postoperative oral hygiene when compared to those who undergo minimally invasive surgery (i.e., laparoscopic surgery), thus oral health care may help to reduce infectious diseases in those patients. However, few reports have shown differences on the oral hygiene status between patients who underwent severely invasive gastrointestinal surgery and those who underwent minimally invasive surgery. In this study we performed a preliminary investigation of changes in bacterial numbers at different sites of the oral cavity, including the tongue dorsum, gingiva of the upper anterior teeth, and palatoglossal arch, as well as dry mouth and tongue coating indices in gastrointestinal cancer patients undergoing surgical treatment who received perioperative oral care. In addition, inflammatory response and nutritional status were investigated in these cases.

## Material and methods

### Subjects

We registered 71 patients (55 men, 16 women; mean age of 72.0 years, ranging from 39 to 97 years) with gastrointestinal cancer who underwent surgery at the Department of Surgery, Takarazuka Municipal Hospital, from November 2015 to February 2017 and received perioperative oral health care. The study design was approved by the Ethics Committee of the Takarazuka Municipal Hospital and all patients signed an informed consent form. Those who suffered from tumor recurrence and received chemotherapy were excluded. Clinical data obtained included patient age, sex, tumor grade, medical history, blood loss volume, operation duration time and hospitalization duration. Tumors were classified using the TNM staging system according to the seventh edition of the American Joint Committee on Cancer Staging Manual^6^. We divided patients based on their surgical treatment into 2 groups, as follows. The minimally invasive surgery group was comprised of those who underwent laparoscopic surgery (n=42; 12 gastric, 22 colon, 8 rectal cancer), while the severely invasive surgery group included patients who underwent open surgical resection (n=29; 20 gastric, 6 colon, 3 rectal cancer). Cephem antibiotics (cefazolin at 1.0 g/day for gastric cancer patients or cefmetazole at 1.0 g/day for rectal cancer patients) were administered to all patients 1-2 days after the operation to prevent surgical site infection. The minimum effective dosage and duration of the antibiotics administrations were determined based on previous reports by the randomized control trial (RCT)^8^ ^,^ ^12^. None of the selected patients had problems associated with denture wearing (i.e., instability, unfitness, fracture). Furthermore, none wore their appliance during the fasting period, during this period it was kept in clean water. Following the fasting period, each participant washed their denture and cleaned it with a soft-bristle brush after taking a meal, resting it in clean water overnight. Clinicopathological factors in the gastrointestinal cancer patients are summarized in Table 1.

**Table 1:** Clinical factors of gastrointestinal cancer patients

Clinical factors	Laparoscopic surgery (42)	Open surgery (29)	p-value
Sex			
Men (55)	34 (61.8%)	21 (38.2%)	0.57
Women (16)	8 (50.0%)	8 (50.0%)	
Age in years			
<65 (14)	12 (85.7%)	2 (14.3%)	0.051
≥65 (57)	30 (52.6%)	27 (47.4%)	
Tumor stage			
Stage I/II (39)	27 (69.2%)	12 (30.8%)	0.096
Stage III/IV (32)	15 (46.9%)	17 (53.1%)	
BMI (kg/m^2^)	22.3±2.9	20.6±2.9	0.28
Number of remaining teeth			
≥20 (31)	18 (58.1%)	13 (41.9%)	0.65
10–19 (16)	11 (68.8%)	5 (31.3%)	
0–9 (24)	13 (54.2%)	11 (45.8%)	
Average number of remaining teeth	15.4±8.7	14.4±9.6	0.71
Denture			
Non-user (28)	19 (67.9%)	9 (32.1%)	0.25
Partial denture (37)	21 (56.8%)	16 (43.2%)	
Full denture (6)	2 (33.3%)	4 (66.7%)	
Smoking			
Non-smoker (37)	17 (45.9%)	20 (54.1%)	0.055
Former smoker (25)	19 (76.0%)	6 (24.0%)	
Current smoker (9)	6 (66.7%)	3 (33.3%)	
Diabetes			
(-) (55)	35 (77.8%)	20 (22.2%)	0.26
(+) (16)	7 (43.8%)	9 (56.3%)	
Hypertension			
(-) (40)	26 (65.0%)	14 (35.0%)	0.38
(+) (31)	16 (51.6%)	15 (48.4%)	
Cardiovascular disease			
(-) (64)	38 (59.4%)	26 (40.6%)	1.0
(+) (7)	4 (57.1%)	3 (42.9%)	
Respiratory disease			
(-) (65)	39 (60.0%)	26 (40.0%)	0.68
(+) (6)	3 (50.0%)	3 (50.0%)	
Cerebrovascular disease			
(-) (65)	38 (58.5%)	27 (41.5%)	1.0
(+) (6)	4 (66.7%)	2 (33.3%)	
Renal disease			
(-) (64)	37 (57.8%)	27 (42.2%)	0.69
(+) (7)	5 (71.4%)	2 (28.6%)	
Operation time (min)	278.2±123.0	309.0±121.3	0.46
Blood loss volume (ml)	116.7±150.8	590.5±556.8	<0.001
Fasting duration (day)	2.9±1.2	4.9±2.1	<0.001
Hospitalization duration (day)	18.6±6.7	31.9±16.9	<0.001

Fisher’s exact test or Mann-Whitney’s U test was used for statistical analysis, with p<0.05 were considered statistically significant

### Oral health care

The protocol for oral care given to the patients was standardized among all dental hygienists who participated in this study. Each patient received self-care instruction on tooth brushing and tongue cleaning, as well as scaling and professional tooth cleaning (PTC) by a dental hygienist at the Oral Surgery Department a day before the operation. Two days after the operation the patients received oral health care and self-care instruction in the inpatient ward by a dental hygienist, then seven days after the operation oral health care such as PTC was given again. In addition, a 0.025% benzalkonium chloride solution was used to disinfect the dorsum of the tongue and upper incisor gingiva.

### Oral bacterial count

The number of oral bacteria in patient samples were determined using a Bacterial Counter (Panasonic Healthcare Co., Ltd., Tokyo, Japan), with the dielectrophoretic impedance change converted to bacterial concentration per ml for each sample [colony-forming units (CFU)/ml]. This device ensures that only viable bacteria are counted. The samples were obtained from the tongue dorsum, gingiva of the upper anterior teeth, and palatoglossal arch, also known as the fauces, using a cotton swab, according to the instructions of the manufacturer. The number of oral bacteria were determined before and after oral health care on preoperative day 1, and again prior to oral care on postoperative days 2 and 7. The samples were collected from 2:00 to 3:00 PM on preoperative day 1, and at 8:00 AM on postoperative days 2 and 7. Furthermore, the same dental hygienist with experience to use the apparatus obtained all samples to maintain reproducibility.

### Evaluation of dry mouth

Changes in dry mouth, also known as xerostomia, and subjective symptoms (i.e., severely dry, moderately dry, slightly dry, moderately wet, fully wet) were evaluated based on interviews performed on preoperative day 1, and on postoperative days 2 and 7. In addition, dry mouth was also classified as normal, light (increased saliva viscosity), moderate (small bubbles observed in saliva), and severe (dry tongue surface without saliva), using a previously reported method^14^.

### Tongue coating index

Tongue coating index (TCI) values were determined on preoperative day 1, and postoperative days 2 and 7. To determine both the extension and thickness of the tongue coating, we used a detailed index previously proposed^29^. With this method, the tongue dorsum is divided into 9 sections, including 1 middle and 2 lateral areas for each of the posterior, middle, and anterior thirds of the tongue. The presence of tongue coating was classified as none (Score 0), light-thin (Score 1, pink color underneath coating visible), and heavy-thick coating (Score 2, no pink color observed) for each section. We obtained a personal tongue coating index by adding the scores for all 9 sections (total score 0-18).

### Clinical examinations

We examined clinical markers such as body temperature (BT), white blood cell (WBC) count, and C-reactive protein (CRP) level to evaluate inflammatory response 1 day before, and 1, 3, and 7 days after the surgery. Increased WBC count is commonly related to inflammatory response, while CRP is a major plasma protein which presents dramatically elevated levels during acute-phase inflammation. In addition, albumin (ALB) level is considered a reliable marker of nutritional status in patients following surgery^24^. We also noted the occurrence of complications after surgery, such as surgical site infection (SSI), anastomotic leakage, and aspiration pneumonia.

### Statistical analysis

We used the Wilcoxon signed-rank test to compare paired data as a non-parametric analysis alternative to a paired t-test, statistically significant *p*-values were considered *p*<0.05. We also used Mann-Whitney’s U test and Fisher’s exact test for statistical analysis.

## Results

### Clinical factors of the gastrointestinal cancer patients

The clinical factors of the gastrointestinal cancer patients are summarized in Table 1. There were no statistical differences for sex, age, tumor stage, body mass index (BMI), number of remaining teeth, or operation duration between the laparoscopic surgery and open surgery groups, however, blood loss volume was significantly higher in the open surgery group (*p*<0.001 Mann-Whitney’s U test). Two of 29 patients in the laparoscopic surgery group and 11 of 42 in the open surgery group received a blood transfusion. In addition, the open surgery group had a significantly longer average length of fasting and hospital stay than the laparoscopic surgery group (4.9±2.1 *vs*. 2.9±1.2 days and 31.9±16.9 *vs*. 18.6±6.7 days, respectively) (*p*<0.001 and *p*<0.001, respectively, Mann-Whitney’s U test).

### Inflammatory response and ALB level in laparoscopic surgery and open surgery groups

**Figure 1:**
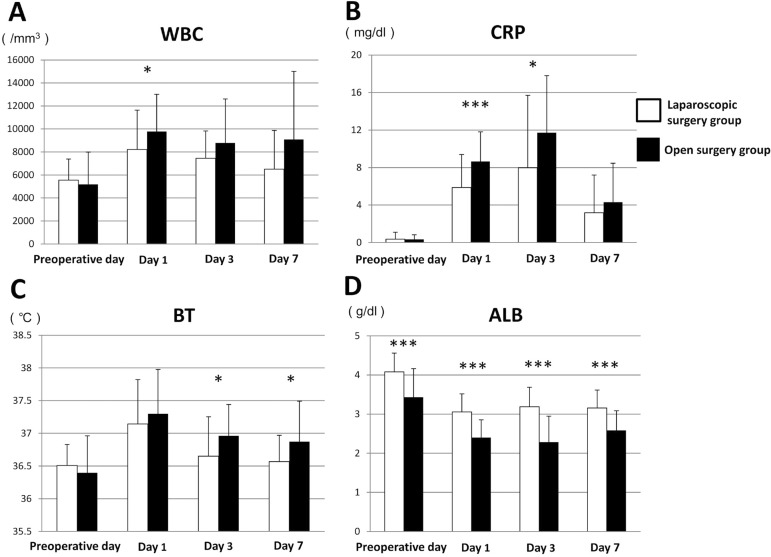
Changes in (A) WBC count, (B) CRP level, (C) BT, and (D) ALB in laparoscopic surgery and open surgery groups. Error bars represent the mean±SD. There were statistically significant differences between the groups. *p<0.05, ***p<0.001

The results of WBC count, CRP level, BT, and ALB are summarized in Table 2. There was a significant difference in mean WBC count between the groups at 1 day after surgery (*p*=0.026, Mann-Whitney’s U test) (Figure 1A). Mean CRP in the laparoscopic surgery group was lower than that in the open surgery group at all moments after surgery. In addition, on postoperative days 1 and 3, the level of CRP was significantly lower in the laparoscopic surgery group (*p*<0.001 and *p*=0.020, respectively, Mann-Whitney’s U test) (Figure 1B). Regarding mean BT, there was a statistically significant difference between the groups on postoperative days 3 and 7 (*p*=0.028 and *p*=0.017, respectively, Mann-Whitney’s U test) (Figure 1C). The mean ALB level was significantly lower in the open surgery group at each moment (*p*<0.001 for each, Mann-Whitney’s U test) (Figure 1D). No patients from either group suffered from any postoperative infectious complications (e.g., SSI, anastomotic leak, aspiration pneumonia).

**Table 2:** Changes in WBC count, CRP level, BT, and ALB

	Perioperative day	Day 1	Day 3	Day 7
**WBC (/mm** ^**3**^ **)**				
Laparoscopic surgery group	5548.6±1833.0	8210.0±3427.4	7451.8±2371.4	6507.1±3363.8
Open surgery group	5185.8±2299.3	9776.3±3229.8	8787.0±3815.4	9089.9±5910.5
**CRP (mg/dl)**				
Laparoscopic surgery group	0.37±0.74	5.87±3.52	7.98±7.72	3.17±4.04
Open surgery group	0.34±0.48	8.67±3.15	11.7±6.08	4.31±4.16
**BT (** ^**o**^ **C)**				
Laparoscopic surgery group	36.5±0.32	37.1±0.68	36.7±0.60	36.6±0.40
Open surgery group	36.4±0.57	37.3±0.68	37.0±0.48	36.9±0.62
**ALB (g/dl)**				
Laparoscopic surgery group	4.08±0.48	3.06±0.46	3.19±0.50	3.20±0.46
Open surgery group	3.43±0.73	2.40±0.46	2.28±0.66	2.58±0.50

### Comparison of number of oral bacteria between laparoscopic surgery and open surgery groups

#### Bacterial number on the tongue dorsum

**Figure 2:**
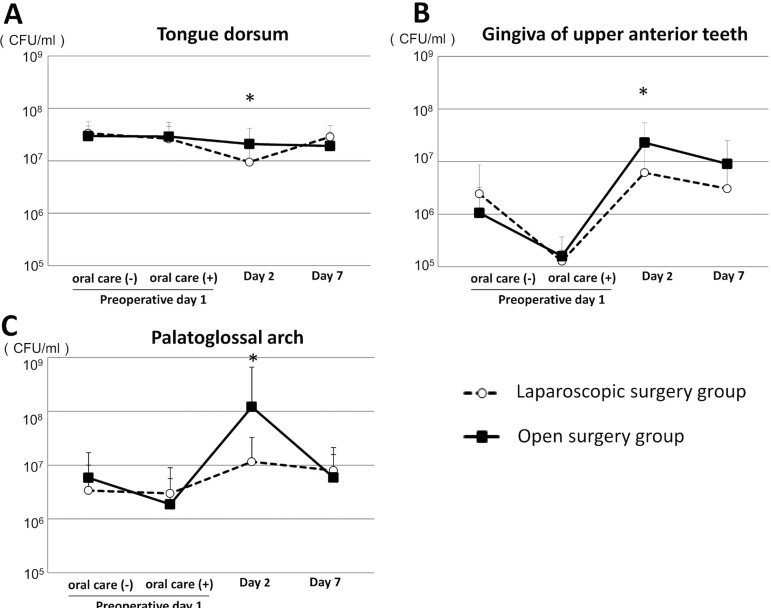
Comparison of oral bacterial counts between laparoscopic surgery and open surgery groups. (A) Numbers of oral bacteria on the dorsum of the tongue. Error bars represent mean±SD. There were statistically significant differences between the groups. *p<0.05; (B) Numbers of oral bacteria on the gingiva of upper anterior teeth; (C) Numbers of oral bacteria on the palatoglossal arch

We examined the number of oral bacteria before and after oral health care performed on preoperative day 1, and prior to oral care on postoperative days 2 and 7 in both groups. There were no significant differences regarding the bacterial count on preoperative day 1, or postoperative days 2 and 7 in the open surgery group. In contrast, the number of bacteria decreased significantly to 10^6.98^ on postoperative day 2 when compared to preoperative day 1 (*p*=0.003, Wilcoxon signed-rank test), and then significantly increased to 10^7.46^ on postoperative day 7 in the laparoscopic surgery group (*p*<0.001, Wilcoxon signed-rank test). In addition, bacterial count was significantly greater on postoperative day 2 in patients who underwent open surgery when compared to the laparoscopic surgery group (*p*=0.033, Mann-Whitney’s U test) (Figure 2A), however, there were no significant differences between the groups before and after oral health care on preoperative day 1, or on postoperative day 7.

#### Bacterial number on the gingiva of upper anterior teeth

The mean bacterial count on preoperative day 1 was 10^6.37^ in the laparoscopic surgery group and 10^6.00^ in the open surgery group, which significantly decreased to 10^5.10^ and 10^5.19^, respectively, immediately after preoperative oral care (*p*<0.001 and *p*=0.001, respectively, Wilcoxon signed-rank test), and then significantly increased to 10^6.79^ and 10^7.36^, respectively, on postoperative day 2 (*p*<0.001, Wilcoxon signed-rank test). In the open surgery group, the mean bacterial count significantly decreased on postoperative day 7 when compared to day 2 (*p*=0.027, Wilcoxon signed-rank test). On postoperative day 2, the mean bacterial count was significantly higher in the open surgery group (P<0.001, Mann-Whitney’s U test) (Figure 2B) and showed a trend to be higher on postoperative day 7, when compared to the laparoscopic surgery group. Furthermore, in the open surgery group, the levels of oral bacteria remained high in the gingiva of the upper anterior teeth even after undergoing postoperative oral health care by a dental hygienist and performing self-care procedures.

#### Bacterial count on the palatoglossal arch

The mean bacterial count on the palatoglossal arch of the open surgery group increased significantly to 10^8.08^ on postoperative day 2 when compared to after oral health care on preoperative day 1 (*p*<0.001, Wilcoxon signed-rank test), and then decreased significantly to 10^6.77^ on postoperative day 7 (*p*=0.001, Wilcoxon signed-rank test). The bacterial count in the laparoscopic surgery group increased to 10^7.06^ on postoperative day 2 when compared to after oral care on preoperative day 1. The mean bacterial count was significantly higher in the open surgery group when compared to the laparoscopic surgery group on postoperative day 2 (*p*<0.001, Mann-Whitney’s U test) (Figure 2C). Both groups presented very similar values on postoperative day 7.

## Comparison of number of oral bacteria between early and advanced tumor stage

**Figure 3:**
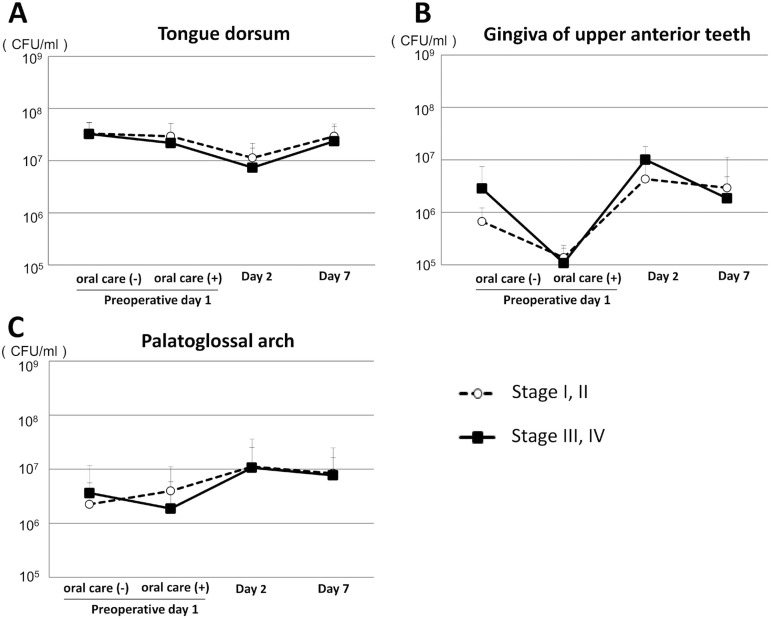
Comparison of oral bacterial counts between early and advanced tumor stage in laparoscopic surgery group. (A) Numbers of oral bacteria on the dorsum of the tongue. Error bars represent mean±SD; (B) Numbers of oral bacteria on the gingiva of upper anterior teeth; (C) Numbers of oral bacteria on the palatoglossal arch

**Figure 4:**
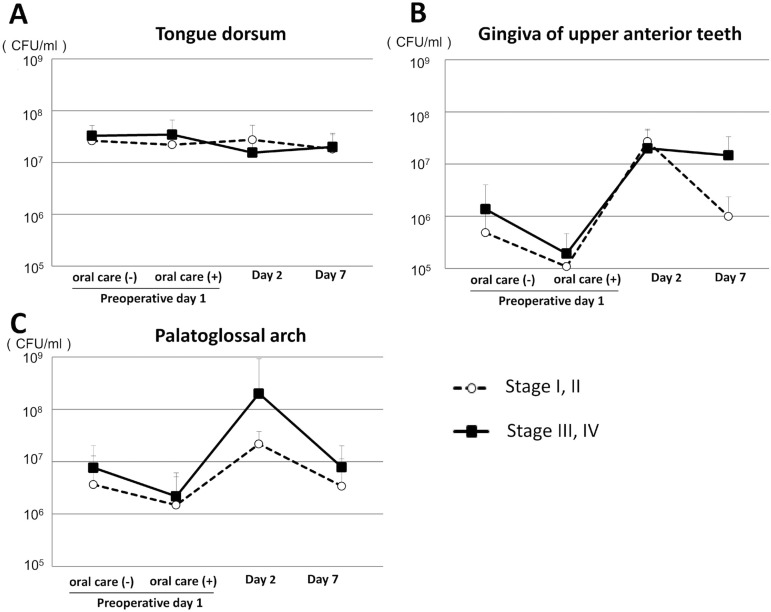
Comparison of oral bacterial counts between early and advanced tumor stage in open surgery group. (A) Numbers of oral bacteria on the dorsum of the tongue. Error bars represent mean±SD; (B) Numbers of oral bacteria on the gingiva of upper anterior teeth; (C) Numbers of oral bacteria on the palatoglossal arch

We compared the number of oral bacteria between patients with early and advanced tumor stage in both groups. For the laparoscopic surgery group there was no significant difference regarding bacterial count between those with a stage I/II and a stage III/IV tumor (Figure 3A, 3B and 3C). In contrast, in the open surgery group the bacterial count in the palatoglossal arch samples obtained on postoperative day 2 was greater in patients with stage III/IV when compared to those with stage I/II gastrointestinal tumors (Figure 4C). In addition, patients with stage III/IV tumors showed increased bacterial count in the gingiva of the upper anterior teeth when compared to those with stage I/II tumors on postoperative day 7, however, the difference was not significant (Figure 4B).

## Comparison of dry mouth between patient groups

**Figure 5:**
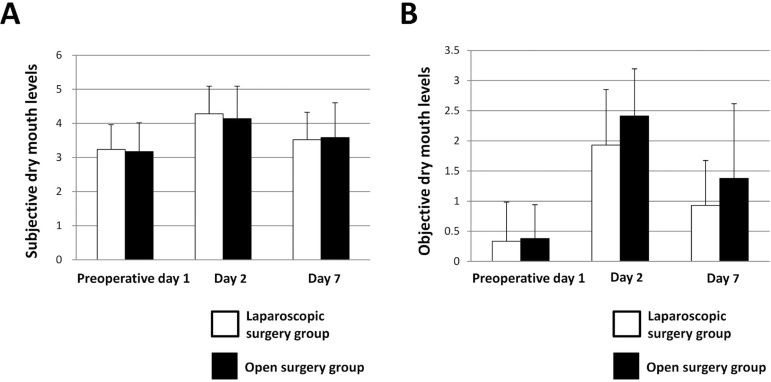
Comparison of dry mouth between laparoscopic surgery and open surgery groups. (A) Subjective dry mouth levels. Error bars represent mean±SD; (B) Objective dry mouth levels. Error bars represent mean±SD

We investigated changes in subjective symptoms related to dry mouth after undergoing oral care on preoperative day 1, as well as on postoperative days 2 and 7. The level of subjective symptoms of dry mouth significantly increased to 4.29±0.81 in the laparoscopic surgery group and to 4.14±0.95 in the open surgery group on day 2 when compared to those on preoperative day 1 (*p*<0.001 and *p*<0.001, respectively, Wilcoxon signed-rank test), and then significantly decreased to 3.52±0.80 and 3.59±1.01 on day 7 (*p*=0.021 and *p*=0.027, respectively, Wilcoxon signed-rank test) (Figure 5A). On the other hand, there was no significant difference regarding the level of dry mouth subjective symptoms between the groups on preoperative day 1, or postoperative days 2 and 7. In addition, dry mouth was objectively evaluated using a previously reported index^14^. There were significant increases to 1.93±0.92 in the laparoscopic surgery group and to 2.41± 0.78 in the open surgery group on postoperative day 2 when compared to the preoperative day 1 results (*p*<0.001 and *p*<0.001, respectively, Wilcoxon signed-rank test), and then significantly decreased to 0.93±0.75 and 1.38±1.24, respectively, on postoperative day 7 (*p*<0.001 and *p*<0.001, respectively, Wilcoxon signed-rank test) (Figure 5B). On postoperative days 2 and 7, the level of dry mouth using the objective method was greater in the open surgery group, however, the differences were not significant.

## Comparison of tongue coating between groups

**Figure 6:**
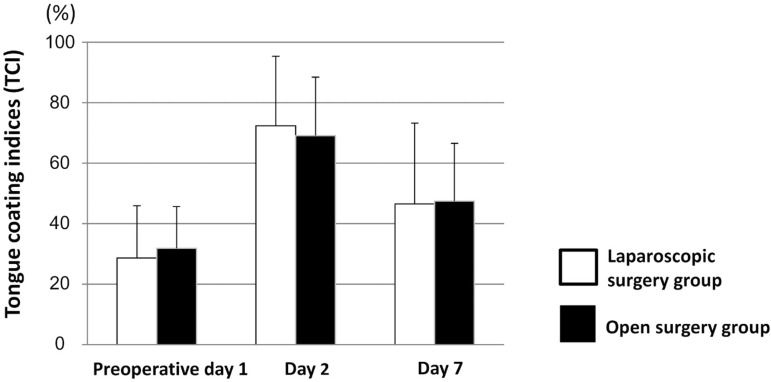
Comparison of tongue coating indices between laparoscopic surgery and open surgery groups. Error bars represent mean±SD

We investigated the relation between the severity of gastrointestinal surgery with tongue coating. The tongue coating index increased to 69.1±19.6% in the laparoscopic surgery group and to 72.3±23.0% in the open surgery group on postoperative day 2 when compared to the values on preoperative day 1 (*p*<0.001 and *p*<0.001, paired t-test), and then significantly decreased to 47.5±19.4% and 46.5±26.7%, respectively, on postoperative day 7 (*p*<0.001 and *p*<0.001, respectively, paired t-test) (Figure 6). However, there was no significant difference in tongue coating indices between the groups at any time point.

## Discussion

In this study, we mainly investigated differences in bacterial number on the gingival mucosa of the upper anterior teeth during the perioperative period and compared our findings between patients with gastrointestinal cancer who underwent severely invasive surgery and those who underwent minimally invasive surgery. There was no great change in number of bacteria on the dorsum of the tongue when compared to samples obtained from the gingiva of the upper anterior teeth and those from the palatoglossal arch. Furthermore, a high oral bacterial count remained on the tongue dorsum after tongue cleaning on preoperative day 1. This could be explained by the anatomical characteristics of the dorsum, which is rough due to its papillae, thus providing a favorable environment for oral bacteria to attach and grow. Furthermore, tongue papillae are relevant as a reservoir of microorganisms, such as anaerobic bacteria, as well as allowing the accumulation of cellular debris (epithelial cells, necrotic leukocytes, among others)^4^.

Inappropriate use of antibiotics may cause disruption of resident oral flora and increase the risk of an antibiotic-resistant strain developing. Antibiotics have effects on the composition of the oral microbiome with temporal variability in healthy individuals^7^. Studies report that the effects of antibiotics administration (i.e., penicillin, 250 mg, 4 times a day for 7 days) on the composition of oral flora were found up to 3 days after therapy, indicating that they are transient when properly administered^23^. Increased temporal variability of the oral and gastrointestinal microbiome was found to be correlated with increased risk of infection in cancer patients receiving chemotherapy^3^ ^,^ ^9^. This observation suggests that the stability of the oral microbiome may play a key role in better outcome for cancer patients.

There are reports that the variability of the oral mucosal microbiome is enhanced by denture use and that increased numbers of disease-related bacterial species (i.e., *Streptococcus mutans*) were found on mucosal surfaces after wearing a partial denture^30^. Another study also reported that dental plaque presented a more diversified microbiome when compared to denture plaque and mucosal plaque obtained from denture wearers, while mucosal plaque obtained from partial denture wearers showed a more significantly diversified microbiome when compared to that from full denture wearers^19^. Furthermore, that study also found that mucosal plaque obtained from dentate individuals possessed a significantly more diversified microbiome when compared to that from edentate individuals. Collectively, these findings indicate that remaining natural teeth as well as denture use may have predominant influences on the diversity of the oral mucosal flora in denture wearers. Furthermore, reports claim that aging is significantly associated with changes in the composition of the oral microbiome in both gingiva and saliva in periodontally healthy individuals who do not use a denture^21^. Thus, aging as well as denture use, may have important roles in the microbiome diversity in the oral cavity.

Combining the mechanical cleaning of the tongue surface, with use of mouth wash and mouth moisturizing gel can effectively reduce bacterial count on the tongue^16^, however, mechanical tongue cleaning alone may not be adequate to remove bacteria harbored in tongue papillae. We note that in this study, tongue coating indices were not associated with the number of bacteria on the tongue surface. This result is consistent with previous studies that investigated the correlation between CFU on the tongue and tongue coating^17^ ^,^ ^22^. During the postoperative fasting period, the tongue papillae can increase on the surface of the tongue in patients who have few opportunities to masticate food. A previous study found a significant association between increased level of tongue coating and tongue motor dysfunction, such as decreased tongue pressure and oral diadochokinesis, in older adults^15^. Therefore, improvement of tongue movement may reduce tongue coating in patients after a period of fasting. Over time, oral bacteria increase in number on the tongue surface, therefore, tongue coating indices may not be correlated to bacterial number immediately after tongue coating formation.

Finding accumulation of “materia alba” and plaque on the gingiva in the upper incisor region in hospitalized patients with poor oral hygiene is very common. Therefore, we investigated bacterial counts in samples from the gingiva in the region of the upper anterior teeth in this study. The bacterial count decreased immediately after undergoing oral health care on preoperative day 1 in both groups. However, on postoperative day 2 the number of bacteria increased in both groups, maintaining high levels in later examinations. These results suggest that bacteria harbored in the gingiva of the upper incisor region can be easily removed by mechanical cleaning, however, they can return easily without additional oral care. Regarding the inflammation of gingival tissues, almost all patients showed no gingival swelling, redness, or bleeding prior to surgery. Evaluation of gingivitis is considered a better assessment of individual oral hygiene practices, because that would reflect exposure to plaque over time. Therefore, using indices of gingival inflammation will be necessary to clarify the correlation between oral hygiene status and bacterial count in a future study.

Saliva has several roles including oral cleaning (i.e., washing away food debris and plaque) and antibacterial functions, which are associated with the regulation of the number of bacteria harbored in the oral cavity^13^. Additionally, the bacterial buffering capacity of saliva varies at different sites in the oral cavity^27^ ^,^ ^28^. Reduced salivary flow may have had effects on the number of oral bacteria during the postoperative fasting period in the patients. Generally, dry mouth is related to an increase in the number of oral bacteria^11^. In our study, the peak of xerostomia was found at 2 days after the operation in both groups, likely because most had no opportunity to gargle during the postsurgical period. Xerostomia and elevated numbers of bacteria in the gingiva of the upper incisor region may be correlated. Furthermore, the decrease in activities of daily living associated with recovery after surgery may have been associated with poor cleaning of the gingiva in the upper incisor region. Some of the open surgery patients remained in bed for 3 or more days after the operation, thus having a longer period of fasting, which may have caused the higher bacterial count in the open surgery group when compared to the count on the laparoscopic surgery group on postoperative days 2 and 7.

Usually, mechanical cleaning should not be applied to the fauces, because it can initiate vomiting reflex. In this study, even though oral health care of the fauces was not performed, there was no distinct difference in bacterial number in the palatoglossal arch samples before and after the operation in the laparoscopic surgery group. On the other hand, the bacterial number in those samples increased significantly in the open surgery group on postoperative day 2, returning to nearly the same number as the preoperative number on postoperative day 7. This increased bacterial count in the open surgery group may have been caused by the worsened dry mouth condition induced by staying in bed for a long duration without oral self-care. Inadequate salivary flow was shown to contribute to severe dry mouth, which led to development of mucositis and colonization of oropharyngeal bacteria in intubated intensive care unit patients^5^. These observations suggest that the improvement of dry mouth may reduce the number of bacteria in the fauces and oropharyngeal regions. Thus, perioperative oral care performed by a dental hygienist is considered as vital to improve the oral health status (i.e., improvement of dry mouth) in patients who have difficulty to perform self-care.

The CRP level was significantly lower in the laparoscopic surgery group on postoperative days 1 and 3 when compared to the open surgery group, indicating that severe damage caused by the open surgery procedures led to distinct differences in CRP between the groups. After undergoing severely invasive surgery, patients with advanced-stage gastrointestinal tumors seem to have a greater increase in bacterial number when compared to those with early-stage tumors. The patients with advanced-stage tumors of the open surgery group showed longer durations of bed resting (2.87±0.77 *vs*. 1.62±1.63 days) and fasting (5.44±2.13 *vs*. 4.15±1.63 days) after the operation than those with early-stage tumors. As a result, those with an advanced-stage tumor likely did not perform self-care and had a decrease in the cleaning function of their saliva, resulting in an increased number of oral bacteria. We note that the decreased ability to perform physical activity soon after the procedure may have been associated with a reduced amount of oral self-care performed by patients treated for an advanced stage tumor. In conclusion, there was a significant difference for oral bacterial count between patients who underwent laparoscopic surgery and those who received abdominal open surgery during the perioperative period, which included oral management treatments. Even when regular oral care was performed, the level of oral bacteria remained high in the gingiva of the upper anterior teeth in the gastrointestinal cancer patients who underwent open surgery. We speculate that the gingiva of the upper incisor region is less susceptible to anti-bacterial functions of saliva when compared to other regions in the oral cavity. Additional procedures are likely needed to effectively reduce the bacterial number in gingiva in that region. Additional studies are required to develop evidence-based perioperative oral health care procedures.
